# Prognostic role of an inflammation scoring system in radical resection of oral squamous cell carcinoma

**DOI:** 10.1186/s12903-022-02261-8

**Published:** 2022-06-08

**Authors:** Meng Wu, Pu Ye, Wei Zhang, Hong Zhu, Huiming Yu

**Affiliations:** 1 Department of Oral and Maxillofacial Surgery, The Affiliated Huaian No.1 People’s Hospital of Nanjing Medical University, Huaian, No. 1, Huanghe West Road, Huaian, 223300 Jiangsu Province China; 2 Department of Pharmacy, The Affiliated Huaian No.1 People’s Hospital of Nanjing Medical University, Huaian, 223300 Jiangsu Province China

**Keywords:** Oral squamous cell carcinoma, F-NLR score, Fibrinogen, Neutrophil–lymphocyte ratio, Prognosis

## Abstract

**Background:**

Inflammatory markers can influence the postoperative prognosis and outcome of malignant tumors. However, the role of inflammatory factors in oral squamous cell carcinoma (OSCC) are still debatable. The primary objective of this investigation was to detect the preoperative blood fibrinogen and neutrophil–lymphocyte ratio (NLR) in OSCC patients and to determine the predictive validity of F-NLR (combined fibrinogen and NLR score).

**Methods:**

A total of 365 patients with oral cancer after surgery were separated into three classes: F-NLR of 2, with hyperfibrinogenemia (> 250 mg/dL) and high NLR (> 3.2); F-NLR of 1, with only one higher index; and F-NLR of 0, with no higher indices. Univariate and multivariate analyses were used to identify risk factors for the demographic and clinical characteristics of patients in the three F-NLR groups. Kaplan–Meier survival analysis was used to assess the prognosis.

**Results:**

Preoperative F-NLR showed a relatively better predictive role in oral cancer prognosis than fibrinogen and NLR alone. Multivariate analysis revealed that F-NLR has the potential to be an independent predictor for OSCC cancer-specific survival (*P* < 0.001). Patients with high scores had a relatively poorer prognosis than those with low scores (*P* < 0.001).

**Conclusions:**

Our findings indicate that blood F-NLR may serve as an independent prognostic factor in OSCC patients.

## Background

The most common cancer in the oral cavity is oral squamous cell carcinoma (OSCC) [1]. OSCC is a highly metastatic tumor, and even patients in early stages have a high rate of recurrence and metastasis. Surgery, radiation, targeted therapy, and chemotherapy are the predominant treatment options for oral cancer, depending on the kind and severity of the disease [2]. Although there have been steady advancements in treatment, OSCC has a poor prognosis when compared to other head and neck cancers. The most likely reason is a combination of variables, including tumor depth, local invasion, nodal involvement, and perineural invasion [3]. Regretfully, these parameters can only be determined after surgical excision and histological investigation. Accordingly, it is critical to determine whether blood indicators can predict OSCC prognosis during the initial clinical evaluation.

Multiple studies have been conducted for years to correlate blood inflammatory factors to predict survival in patients with OSCC. Cancer cell adhesion is facilitated by systemic inflammation, which is signaled by the increase in circulating neutrophils at the front line of defense [4–6]. An important feature of cancer is the ability to evade killing by the immune system [7]. Studies have shown that tumor-infiltrating lymphocytes (TILs) play a key role in tumor progression in the tumor immune microenvironment, including that of OSCC [8, 9]. Multiple studies have confirmed that elevated levels of TILs are associated with good prognosis in OSCC [10, 11]. Lymphocytes, particularly cytotoxic lymphocytes, influence cancer progression and cancer treatment by eliminating tumor cells [12, 13]. The available evidence suggests that increased pretreatment neutrophil and lymphocyte numbers are correlated with a poor cancer outcome [14–16].

Fibrinogen is a protein that promotes inflammation and is produced in the liver as a result of interleukin-6 and IL-1b activation [17, 18]. Fibrinogen is converted to fibrin by activated thrombin in the coagulation cascade and can regulate the development of malignant tumors [19]. Recent research has confirmed that plasma fibrinogen levels play an oncogenic role in a variety of human cancers [20–23]. NLR (neutrophil–lymphocyte ratio) has become a prognostic indicator for many malignant tumors, and a high NLR often shortens the survival of patients [24–26]. Alternatively, NLR could be used as an independent prognostic indicator in patients with OSCC [27]. A recent meta-analysis of 17 studies with 4597 patients showed that pre-treatment NLR correlates with a statistically significant decrease of OS in head and neck squamous cell carcinoma patients [28]. The generation of inflammatory cytokines/chemokines by neutrophils facilitates the occurrence and development of tumors by creating a suitable tumor microenvironment [29]. Currently, there is no data on the combined parameters of plasma fibrinogen and NLR in the preoperative environment to predict the prognosis of OSCC.

The objective of this research was to determine the prognostic significance of F-NLR (combined fibrinogen and NLR score) in patients with OSCC. This work will generate fresh insight into the clinical efficacy of a composite score based on F-NLR as a prognostic factor of OSCC.

## Methods

### Materials and methods

A total of 365 individuals with OSCC treated with radical resection at the Department of Oral and Maxillofacial Surgery, The Affiliated Huaian No.1 People’s Hospital of Nanjing Medical University from February 2014 to November 2019 were enrolled in this retrospective analysis. The eligibility criteria included patients who had (1) a confirmed pathological diagnosis of OSCC; (2) no history of cancer; (3) no distant metastasis; (4) standard surgical approach: including primary tumor resection and neck dissection; (5) available clinical data and follow-up data; (6) no other conditions that could cause the blood values to change; and (7) no adjuvant radiotherapy or chemotherapy preoperatively. Patients with missing or partial data were excluded from the research. Preoperative medical and blood tests, as well as other necessary examinations, were performed on these patients to aid in the proper planning of surgery.

### Blood assessment for determination of fibrinogen and NLR

One week before the start of treatment, blood samples were taken. The blood samples were immediately sent to the laboratory for testing of relevant indicators. Neutrophils and lymphocytes were measured using a SYSMEX Analyzer CS5100, Japan. Plasma fibrinogen concentrations were detected by a SYSMEX analyzer XN-9000, Japan. The measured indices were recorded, and the NLR is indicated as the neutrophil count/lymphocyte count.

### Calculation of prognostic scores

The cutoff threshold for fibrinogen based on our study was 250 mg/dL. The cutoff threshold of NLR based on our data was 3.2. The F-NLR score was classified into three groups: F-NLR of 2, with hyperfibrinogenemia (> 250 mg/dL) and high NLR (> 3.2); F-NLR of 1, with only one higher index; and F-NLR of 0, with neither hyperfibrinogenemia nor high NLR.

### Follow-up

Follow-up was performed monthly for the first 6 months and then every 6 months via telephone follow-up or clinical follow-up. From the time of surgery to death because of OSCC, the duration of cancer-specific survival (CSS) was recorded.

### Statistical analysis

Univariable and multivariable analyses were used to evaluate clinical factors for CSS. Categorical data were analyzed by the chi-square test or Fisher’s exact test. Continuous data were compared by the Mann–Whitney U test. Multivariate Cox regression analysis and Kaplan–Meier analysis were utilized to assess CSS of OSCC. All data analyses were performed with SPSS (IBM SPSS 22.0, SPSS Inc.). Statistical tests were two-sided and considered significant with a *P value* < 0.05.

## Results

### Demographic data

A total of 365 patients who were eligible to participate were included in this study. The age of treatment ranged from 21 to 90 years, with males averaging 64 years and females averaging 63 years. A total of 174 (47.7%) of the patients were females, whereas 191 (52.3%) were males. Tumor size ≤ 4 cm (80.0%) and no lymph node metastases (78.4%) were the most common clinicopathological characteristics (Table 1).

**Table 1:** Comparison of clinical characteristics of the enrolled subjects (n = 365)

Variables	Patients (n, %)
*Sex*	
Male	191 (52.3)
Female	174 (47.7)
*Age*	
< 60	107 (29.3)
≥ 60	258 (70.7)
*Currently smoking*	
No	327 (89.6)
Yes	38 (10.4)
*Currently drinking*	
No	336 (92.1)
Yes	29 (7.9)
*NLR*	
≤ 3.2	274 (75.1)
> 3.2	91 (24.9)
*Fibrinogen (mg/dl)*	
≤ 250	129 (35.3)
> 250	236 (64.7)
*F-NLR*	
0	100 (27.4)
1	203 (55.6)
2	62 (17.0)
*PLR*	
< 150	67 (18.4)
≥ 150 ≤ 300	285 (78.0)
> 300	13 (3.6)
*MPV (fl)*	
< 10	315 (86.3)
≥ 10	50 (13.7)
*Tumor size*	
T1–T2	292 (80.0)
T3–T4	73 (20.0)
*Cervical node metastasis*	
N0	286 (78.4)
N1	50 (13.7)
N2	29 (7.9)
*Cancer subsites*	
Lip	30 (8.2)
Gingval	104 (28.5)
Buccal	112 (30.7)
Mouth floor	15 (4.1)
Tongue	104 (28.5)
*LVI*	
Negative	263 (72.1)
Positive	102 (27.9)
*PNI*	
Negative	356 (97.5)
Positive	9 (2.5)

*F-NLR* the combined fibrinogen and NLR score, F-NLR of 2, with hyperfibrinogenemia (> 400 mg/dL) and high NLR (> 2.5), F-NLR of 1, with only one higher index, and F-NLR of 0, with neither hyperfibrinogenemia nor high NLR, *NLR* Neutrophil-to-lymphocyte ratio, *MPV* mean platelet volume, *PNI* perineural invasion, *LVI* lymphatic and vascular invasion

### ROC analysis

**Fig. 1:**
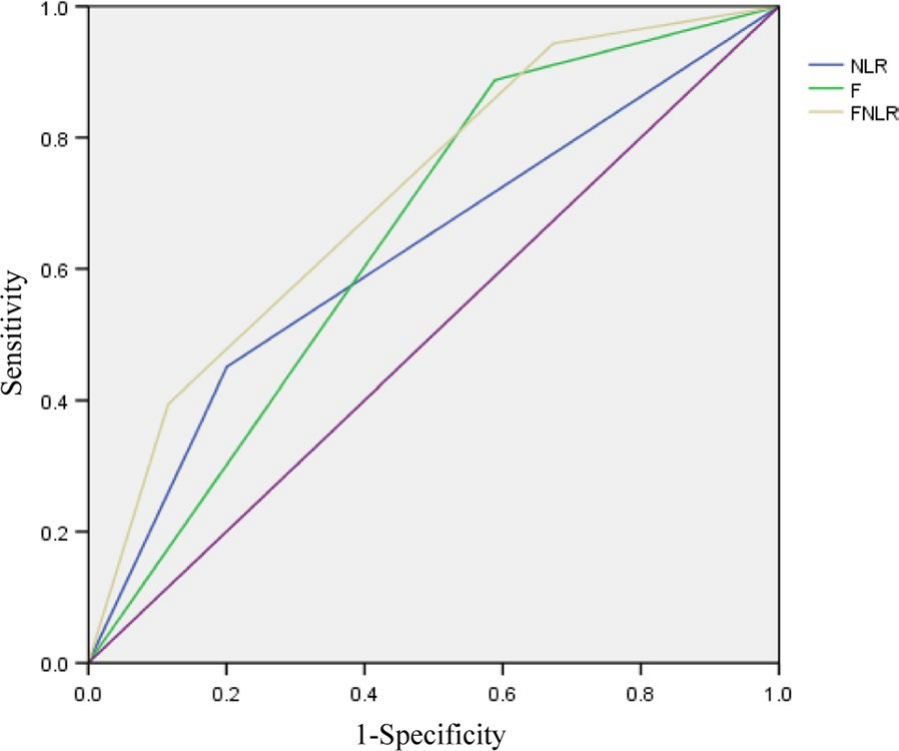
ROC curve for fibrinogen, NLR and F-NLR in OSCC patients. Fibrinogen, NLR, and F-NLR were the test variables, and CSS was the state variable. The areas under the curve are 0.649, 0.625 and 0.713, respectively. *ROC* receiver operating characteristic, *F-NLR* combined fibrinogen and NLR score, *NLR* neutrophil-to-lymphocyte ratio, *CSS* cancer-specific survival, *OSCC* oral squamous cell carcinoma, *F* fibrinogen

We identified the survival prediction values of fibrinogen, NLR and F-NLR using ROC curve analysis. Figure 1 shows the ROC curves and AUC results: the curve of fibrinogen (AUC = 0.649, 95% CI 0.585–0.741, and cutoff value = 250 mg/dL); the curve of NLR (AUC = 0.625, 95% CI 0.548–0.702, and cutoff value = 3.2); and the curve of F-NLR (AUC = 0.713, and 95% CI 0.649–0.788). Preoperative F-NLR showed a relatively better predictive role in oral cancer prognosis than fibrinogen and NLR alone (Fig. 1).

### Correlation of fibrinogen and NLR with clinicopathological characteristics

Patients were divided into three groups according to F-NLR (0, n = 100; 1, n = 203; 2, n = 62). The clinicopathological characteristics of the patients in the three groups are listed in Table 2. Higher F-NLRs were associated with tumor size (*P* = 0.0301), cervical node metastasis (*P* = 0.0039) and lymphatic and vascular invasion (LVI) (*P* = 0.0009).

**Table 2:** The clinicopathological characteristics stratified by the F-NLR score

Characteristics	F-NLR 0 (n = 100)	F-NLR 1 (n = 203)	F-NLR 2 (n = 62)	*P*-value
*Age*				**0.0436**
< 60	32	65	10	
≥ 60	68	138	52	
*Sex*				0.3646
Male	47	108	36	
Female	53	95	26	
*Currently smoking*				0.2439
No	87	181	59	
Yes	13	22	3	
*Currently drinking*				0.8427
No	91	187	58	
Yes	9	16	4	
*PLR*				0.5971
< 150	21	36	10	
≥ 150 ≤ 300	77	160	48	
> 300	2	7	4	
*MPV (fl)*				0.4345
< 10	90	173	52	
≥ 10	10	30	10	
*Tumor size*				**0.0301**
T1–T2	89	155	48	
T3–T4	11	48	14	
*Cervical node metastasis*				**0.0039**
N0	81	167	38	
N1	15	20	15	
N2	4	16	9	
*Cancer subsites*				0.8078
Lip	8	15	7	
Gingival	24	64	16	
Buccal	32	58	22	
Mouth floor	4	8	3	
Tongue	32	58	14	
*LVI*				**0.0009**
Negative	86	133	44	
Positive	14	70	18	
*PNI*				0.8873
Negative	98	198	60	
Positive	2	5	2	

Statistically significant values are shown in bold

*F-NLR* the combined fibrinogen and NLR score, *NLR* Neutrophil-to-lymphocyte ratio, *MPV* mean platelet volume, *PNI* perineural invasion, *LVI* lymphatic and vascular invasion

### Risk factors for the prognosis of OSCC

**Fig. 2:**
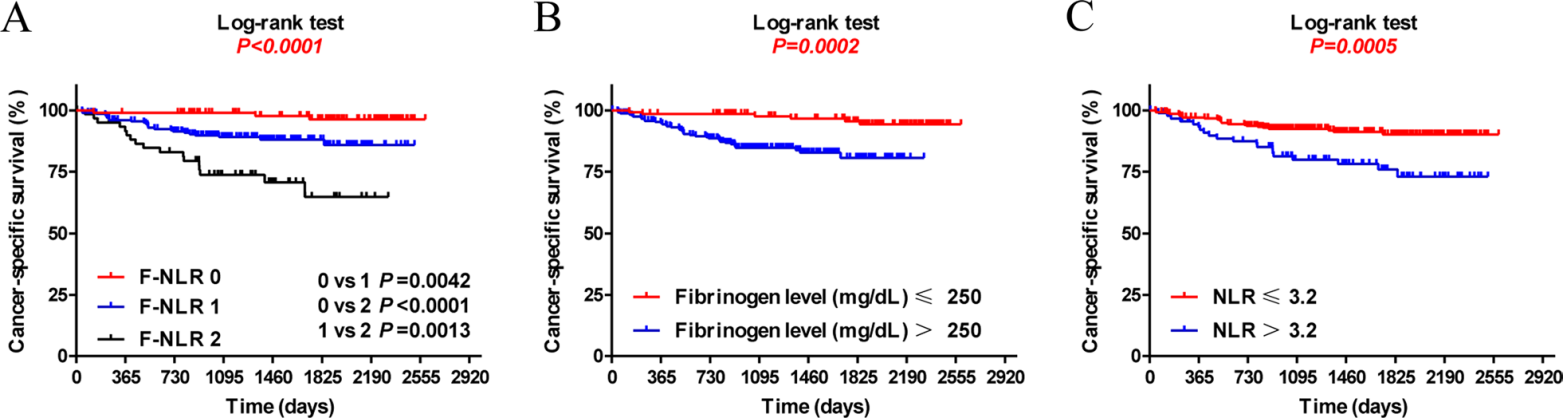
Kaplan–Meier curves for CSS according to F-NLR (**A**), fibrinogen (**B**), and NLR (**C**) in OSCC patients. *F-NLR* combined fibrinogen and NLR score, *NLR* neutrophil-to-lymphocyte ratio, *CSS* cancer-specific survival, *OSCC* oral squamous cell carcinoma

Fourteen clinicopathological parameters were included in the univariate analyses, and nine significant parameters for univariate analysis were included in the multivariate analyses (Table 3). Multivariate analysis showed that F-NLR was an independent prognostic factor for cancer-specific survival (HR for F-NLR 1 and F-NLR 2: 26.566 and 3.895; 95% CI 5.589–126.258 and 1.321–11.485; *P* < 0.001 and *P* = 0.014, respectively). Patients with high F-NLR had a poor prognosis (Fig. 2).

**Table 3:** Univariate and multivariate analyses of prognostic factors in 365 patients with OSCC

Variable	Univariate survival analysis			Multivariate survival analysis		
	Hazard ratio	95% CI	*P*-value	Hazard ratio	95% CI	*P*-value
Sex	1.054	0.662–1.679	0.823			
Currently smoking	0.931	0.426–2.031	0.857			
Currently drinking	0.869	0.350–2.157	0.761			
Age	1.858	1.036–3.333	**0.038**	1.930	1.051–3.546	**0.034**
Tumor size	3.355	2.024–5.555	** < 0.001**	2.469	1.408–4.329	**0.002**
Cervical nodal metastasis						
N0	Ref			Ref		
N1	4.807	2.739–8.403	** < 0.0001**	2.688	1.373–5.263	**0.004**
N2	2.941	1.344–6.410	**0.007**	3.663	0.686–3.787	0.273
Cancer subsites						
Lip	Ref					
Gingval	0.788	0.267–2.320	0.665			
Buccal	1.194	0.646–2.206	0.572			
Mouth floor	1.252	0.686–2.287	0.464			
Tongue	0.765	0.178–3.286	0.719			
NLR	2.618	1.640–4.180	** < 0.001**	1.978	1.194–3.274	**0.008**
Fibrinogen (mg/dL)	8.128	3.641–18.144	** < 0.001**	5.301	2.426–11.581	** < 0.001**
MPV	2.707	1.577–4.645	** < 0.001**	1.804	1.041–3.127	**0.035**
PLR						
< 150	Ref			Ref		
≥ 150 ≤ 300	6.774	2.913–15.750	** < 0.001**	2.468	1.053–5.783	**0.037**
> 300	6.758	3.406–13.406	** < 0.001**	2.388	1.152–4.951	**0.019**
F-NLR						
0	Ref			Ref		
1	19.989	6.847–58.359	** < 0.001**	26.566	5.589–126.258	** < 0.001**
2	2.621	1.612–4.261	** < 0.001**	3.895	1.321–11.485	**0.014**
LVI	2.290	1.401–3.744	**0.001**	2.079	1.234–3.496	**0.006**
PNI	2.390	0.750–7.616	0.141			

Statistically significant values are shown in bold

*F-NLR* the combined fibrinogen and NLR score, *NLR* Neutrophil-to-lymphocyte ratio, *MPV* mean platelet volume, *PNI* perineural invasion, *LVI* lymphatic and vascular invasion, *OSCC* oral squamous cell carcinoma

**Fig. 3:**
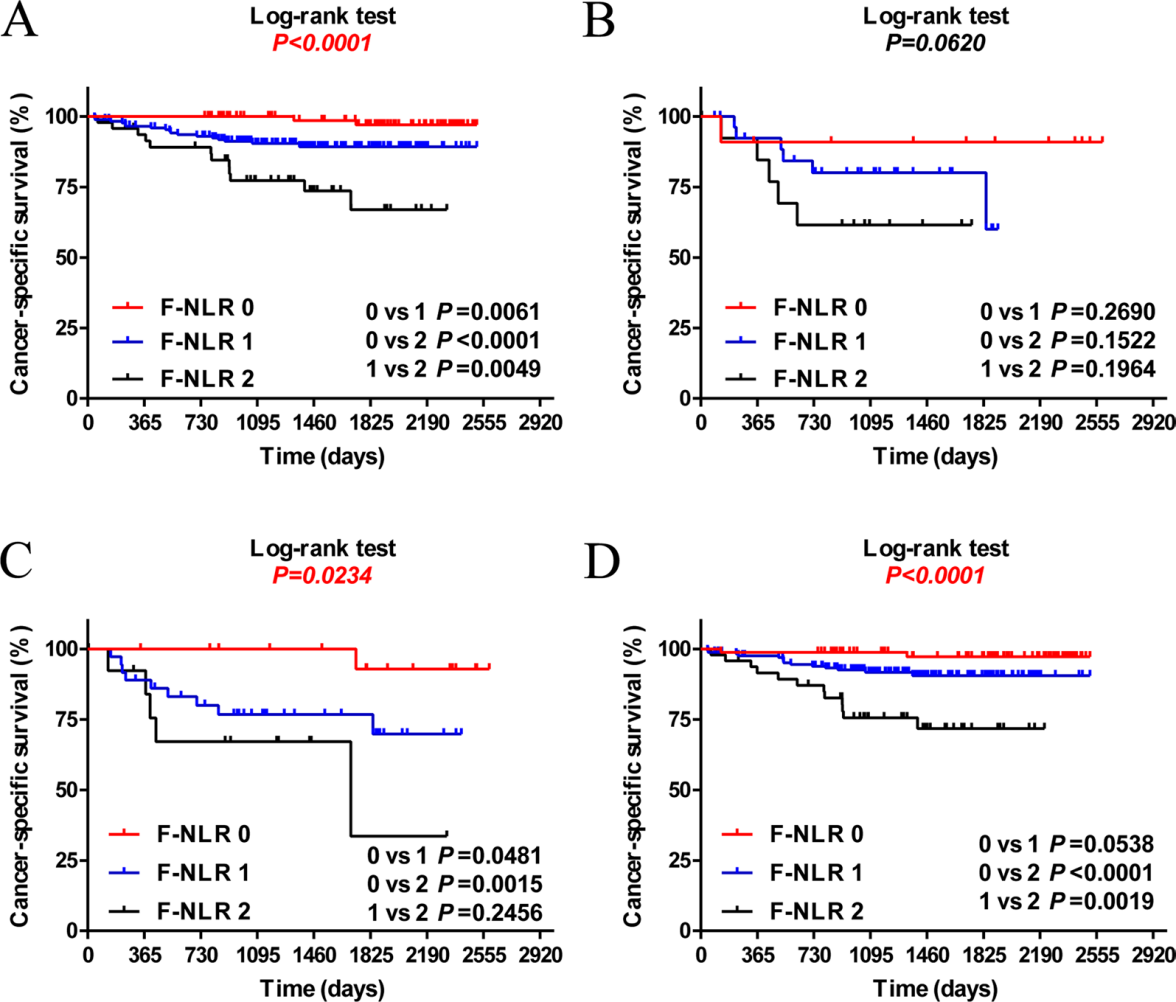
CSS based on F-NLR in OSCC patients with T1–T2 tumors (**A**), patients with T3–T4 tumors (**B**), patients with lymphatic metastasis (**C**) and patients without lymphatic metastasis (**D**). *F-NLR* combined fibrinogen and NLR score, *NLR* neutrophil-to-lymphocyte ratio, *CSS* cancer-specific survival, *OSCC* oral squamous cell carcinoma

In a stratified analysis based on tumor size and lymph node metastasis, the results showed that the prognostic value of F-NLR was maintained for T1–T2 (*P* < 0.0001, Fig. 3A), N (−) (*P* = 0.0234, Fig. 3C) and N (+) (*P* < 0.0001, Fig. 3D) tumors, except for T3–T4 (*P* = 0.0620, Fig. 3B) tumors.

## Discussion

Oral cancer is the world's 11th most prevalent cancer [30]. Diagnosis at the early stage is critical for improving patient survival rates. When identified early, the survival probability is approximately 80–90% [31]. Therefore, it is critical to assess patients' prognostic variables before surgery. Plasma biomarkers have great potential for predicting tumor recurrence, because they can help surgeons make more individualized treatment plans [32]. Furthermore, these markers can be collected through standard blood testing prior to surgery, which is both cost effective and convenient.

Patients with cancer frequently have a better prognosis when there is no inflammation. The detection of circulating cell components is the most common method for measuring the degree of activation of the systemic inflammatory response [33]. For several common peripheral blood-derived inflammation scores, neutrophils play an active role in promoting tumor progression [34]. TILs play important roles in the progression of OSCC. Three main immunophenotypes are currently recognized according to the distribution of T cells in the tumor: immune-inflamed, immune-excluded, and immune-desert [35]. Immune-inflamed tumors are characterized by a dense infiltration of immune cells in close proximity to tumor cells. Immune-excluded tumors are characterized by the presence of large numbers of immune cells that do not penetrate the tumor parenchyma but remain in the stroma. The immune-desert phenotype is characterized by an almost complete absence of immune cells in the tumor parenchyma or stroma. Troiano et al. demonstrated that squamous cell carcinoma of the tongue patients with an immune-desert phenotype had lower survival [36]. Based on the above results, lymphocytes can significantly inhibit the proliferation of tumors [37]. Therefore, the NLR can serve as a reliable tumor prognostic factor. Numerous studies have confirmed that NLR is a prognostic factor for a variety of benign and malignant tumors and a representative indicator of tumor-associated inflammation, and NLR has been shown to have predictive value for oral cancer [38–41]. Activation of the coagulation cascade has a substantial effect on the development of cancer, and fibrinogen has been shown to be one of the regulators of systemic inflammation and tumor progression [42, 43]. These studies have laid a solid foundation for the next step to explore the role of inflammatory factors in the prognosis of oral cancer. In this study, patients with advanced tumors tended to have a high fibrinogen level and high NLR. In addition, high fibrinogen and high NLR were found to be important indicators for predicting clinical outcome in this study (*P* < 0.001 and *P* = 0.008). The results of our study are consistent with those of previous meta-analyses, showing that high NLR significantly reduces the survival time of head and neck squamous cell carcinoma patients [28]. We propose the following mechanism of action: Fibrinogen may function by forming a protective framework that facilitates tumor migration, invasion, and angiogenesis. Tumor cells create an inflammatory state in the body and accelerate the production of fibrinogen in the liver, thus forming a vicious cycle.

We also confirmed that age, tumor size, and cervical nodal metastasis are independent prognostic factors for oral squamous cell carcinoma, as previously reported by Broglie et al. [44] and Patel et al. [45]. Interestingly, the multivariate analysis showed that different MPV (mean platelet volume) levels can also significantly affect the prognosis of oral cancer. MPV is an indicator of platelet activation and has been found to be a prognostic indicator in a variety of cancers [46]. The value of MPV as a prognostic indicator may be that activated platelets promote the secretion of cytokines, such as platelet-derived endothelial cell growth factor, which accelerates tumorigenesis [47]. In a study observed over 30 years, Zanoni et al. found that perineural invasion (PNI) and lymphatic and vascular invasion (LVI) were independent prognostic factors of OSCC [48]. However, unlike the previous study, our data analysis showed that only LVI was prognostic factor for OSCC, but PNI was not statistically different. The reason may be that we only have nine PNI positive patients, and analyses with larger sample sizes require further validation.

The new scoring system (F-NLR) is a good predictor for the prognosis of a variety of malignancies. Wang et al. proved that F-NLR can independently predict the prognosis of patients with non-small-cell lung cancer [49]. Data from Felice's studies showed that F-NLR is substantially related to worse survival results in individuals with anal canal cancer [50]. The above research has established that the F-NLR is a reliable scoring system for assessing malignant tumors. No detailed investigation of the application prospects of the F-NLR scoring system in oral cancer has been performed. Therefore, this retrospective study was performed to identify the association of F-NLR with prognosis after radical resection of oral cancer.

For predictive analysis following radical excision of oral cancer, we separated the patients with different F-NLR into three independent groups. The results suggested that F-NLR, as predicted, can identify more individuals with a worse prognosis than fibrinogen or NLR alone. This finding demonstrates that F-NLR may be more reliable than individual scores. The median survival times were 62 months (scores of 0) and 41 months (scores of 1–2), respectively. Patients with high F-NLR had a substantially poorer prognosis than those with low scores. Meanwhile, these data show that F-NLR is connected to tumor development and aggressiveness. In our subgroup analyses, F-NLR was a good prognostic factor; for example, in patients with T1–T2 tumor sizes and different lymph node metastases, the F-NLR scoring system showed a good prognostic effect.

This study has some limitations. First, a single-center retrospective study may lead to selection bias. Second, the short follow-up period was not sufficient to further assess the survival of patients. Consequently, a more comprehensive multicenter prospective study is needed to confirm that F-NLR does independently predict prognosis in patients with OSCC.

According to the findings of this study, F-NLR has clinical promise as a low-cost prognostic factor in patients with OSCC.

## Conclusion

This study suggests that the F-NLR is an independent prognostic indicator for the survival rate of OSCC.

## Data Availability

The datasets generated and analyzed during the current study are not publicly available, but are available from the corresponding author on reasonable request.

## Abbreviations

OSCC: Oral squamous cell carcinoma
F-NLR: Combined fibrinogen and NLR score
NLR: Neutrophil-to-lymphocyte ratio
AUC: Area under the receiver operating characteristic curve
CI: Confidence interval
ROC: Receiver operating characteristic
CSS: Cancer-specific survival
TILs: Tumor-infiltrating lymphocytes
MPV: Mean platelet volume
PNI: Perineural invasion
LVI: Lymphatic and vascular invasion
